# Transfusion rates and disease spectrum in neonates treated with blood transfusion in China

**DOI:** 10.1097/MD.0000000000019961

**Published:** 2020-05-01

**Authors:** Yang Sun, Ting Ma, Wen-hua Wang, Qin Zhang, Zhen-ai Jin, Jiang-Cun Yang

**Affiliations:** a Department of Transfusion Medicine; b Department of Neonatology, Shaanxi Provincial People's Hospital, Xi’an 710068; c Department of Pediatrics, Affiliated Hospital of Yanbian University, Yanji 133000, China.

**Keywords:** blood transfusion, blood transfusion rate, disease spectrum, neonates

## Abstract

This study aimed to investigate blood transfusion rates and spectrum of diseases in hospitalized neonates treated with blood transfusion in China to provide supporting data for future studies on neonatal blood transfusion.

Data on hospitalized neonates were obtained from more than 100 experts from the Department of Neonatology of 55 hospitals in China between January 1, 2012 and December 31, 2016, using a standardized survey. A statistical analysis was conducted to evaluate the data collected, including the blood transfusion rates, blood component transfused, spectrum of diseases, and spectrum of major diseases.

Between 2012 and 2016, 541,128 neonates were hospitalized in the 55 hospitals surveyed. There were 70,433 neonates who received blood transfusion, with an average transfusion rate of 13.02%. The rates of red blood cell transfusion, platelet transfusion, and plasma transfusion were 9.44%, 0.66%, and 4.77%, respectively. The neonatal blood transfusion rate was 17.99% in Northeast China, 9.74% in Northwest China, and between 10.60% and 16.22% in other regions. The neonatal blood transfusion rate was 12.3% in general hospitals and 13.8% in women and children's hospitals. The top 10 diseases identified in hospitalized neonates treated by blood transfusion were, in rank order, as follows:prematurity,pneumonia, hyperbilirubinemia, bacterial sepsis, respiratory distress syndrome, anemia, hemolytic disease, asphyxia, hemorrhage, and necrotizing enterocolitis.

The neonatal blood transfusion rate in China is 13.03%.The rank order in the disease spectrum of the hospitalized neonates and that in hospitalized neonates treated with blood transfusion are different.

## Abstract 2

Supplemental Digital Content is available in the text

## 1 Introduction

Neonatal transfusion therapy is used for the treatment of anemia, thrombocytopenia, and diseases associated with coagulation disorders of different etiologies. Since the “two-child policy” has come into effect in China, the number of women at advanced maternal age with high-risk pregnancy has increased rapidly, leading to an increase in the number of preterm neonates, low-birth-weight(LBW) neonates, and neonates with postnatal defects. The two-child policy is a family planning policy implemented in China, which means that couples who meet the specified conditions are allowed to have “two children” since January 1, 2016. The couples are allowed to have only “one children” before that time. Blood transfusion therapy is particularly important for the treatment of LBW or very-low-birth- weight(VLBW) neonates, in which the transfusion rate during hospitalization can reach more than 80%.^[1]^ However, since there are no big data from neonatal transfusion research in China to aid in the formulation of national neonatal transfusion guidelines, the decisions for neonatal transfusion therapy in the clinical settings in China are mainly made on empirical basis or with reference to the transfusion guidelines from foreign countries. Some countries, such as the United Kingdom, the United States, Italy, and Australia, have developed guidelines or expert consensus on neonatal transfusion therapy that provide guidance on transfusions for neonatal thrombocytopenia and neonatal anemia.^[2–7]^ However, the recommendations are inconsistent, and treatment decisions are often still based on clinical experience.^[8]^

This study utilized a multicenter and a combined retrospective and prospective survey design to investigate the status of neonatal blood transfusion in China. The data on neonatal blood transfusions obtained can provide an evidence-based medical basis for the development of neonatal blood transfusion treatment guidelines in China.

## 2 Methods

### 2.1 Study population

(1) Study population. Between January 1, 2012 and December 31, 2016, all neonates hospitalized in the Department of Neonatology of 55 hospitals nationwide were included, and all studies that involve human subjects were approved by the ethics committee of Shaanxi Provincial People's Hospital.

The 55 selected hospitals should match any of the following conditions:

(1) the number of neonates department beds are greater than 20 in the tertiary general hospital.
(2) Children's hospital or maternal and child health hospital.

The hospitals were nationwide collected on the net, and the 55 participating hospitals were extracted at random from them according to the regions.

(2) Patient groupings.

(1) By region: data were grouped based on the administrative regions of China: Northeast China, North China, East China, Central China, South China, Northwest China, and Southwest China. Data from Hong Kong, Macau, and Taiwan were not collected.
(2) By hospital type: the hospital type was divided into general hospitals and women and children's hospitals.
(3) By quartile: data were grouped into quartiles based on the different number of beds in the Department of Neonatology of the hospitals: 1 to 48 beds (within 25%), 49 to 64 beds, 65 to 90 beds, and >91 beds (75% or above).

### 2.2 Data collection

(1) Collection method. A standardized data collection table was developed and used to collect the basic information and data related to blood transfusion of the hospitalized neonates in the Department of Neonatology of 55 hospitals between 2012 and 2016. The data regarding the top 10 diseases identified in the hospitalized neonates and top 10 diseases identified in the hospitalized neonates treated with blood transfusion each year were collected from the medical record department or information center of each hospital. The International Classification of Diseases (ICD) codes were used as the selection criteria for the collection of disease-related information: the four-digit ICD codes (category) were used for the primary screening of disease and for determining the disease classification.
(2) Determination of the number of patients who received blood transfusions. (1) the number of patients who received blood transfusions: a single blood transfusion therapy or multiple blood transfusion therapies during hospitalization were recorded as one transfusion. (2) The number of patients who received blood component transfusions: transfusions of different blood components during hospitalization were calculated separately, for example, one red blood cell suspension transfusion and one plasma transfusion separately.
(3) Quality control. Data collection and joint auditing were conducted by one neonatologist and one blood transfusion technician in each participating hospital. All participants received a standardized training. The data acquisition were carried out by the participating hospitals according to the questionnaire. The carried data were sent to the Shaanxi Provincial People's Hospital, where the experts of the research group performed data cleansing and quality control of the collected data.

### 2.3 Disease classification

(1) Assignment of disease. Data of the top 10 diseases identified in the hospitalized neonates and the top 10 diseases identified in those treated with blood transfusion in each hospital were collected and sorted as follows:

(1) The diseases were grouped according to the ICD.
(2) Assignment of disease: the top 10 assigned diseases in each hospital were assigned a value between 1 and 10 (10, 9, 8, …, 1 points) according to their rank.
(3) To exclude the impact of hospital size and the number of discharged patients in the Department of Neonatology on the overall disease ranking, data weighting was conducted based on the disease assignments in each hospital, the number of discharged patients in the Department of Neonatology, and the total number of discharged neonates in all participating units.

The scores (*r*_s_) of the top 10 diseases in this unit relative to the diseases in all participating units in the nation was calculated as follows: 
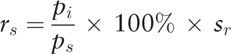


where *r*_s_ represents the scores of the disease, *p*_i_ as number of discharged neonatal patients in each participating unit during the survey reference period, *p*_s_ as total number of discharged neonatal patients in all participating units during the survey reference period, and *s*_r_ as disease type in discharged neonatal patients in each participating unit during the survey reference period. Each disease was assigned a value according to its rank (1st = 10 points, 2nd = 9 points, etc.).

(2) Rank order in the disease spectrum. The r_s_ of the same disease from the 55 hospitals were classified and summed to obtain a total score for each disease. Finally, a disease spectrum was obtained by ranking different diseases according to their total scores.

### 2.4 Statistical analysis

All data were analyzed using the IBM SPSS 24.0 statistical software, and categorical data were expressed as rates and ratios.

## 3 Results

### 3.1 Background information of the 55 hospitals

The 55 hospitals included in this study were located across 7 administrative regions, 21 provinces, 2 direct-controlled municipalities, and 41 cities in China. A total of 106,538 beds were in use in the 55 hospitals, including 3924 beds in the Department of Neonatology. Of the 55 hospitals, 17 were women and children's hospitals, while 38 were general hospitals (Table 1).

**Table 1:**
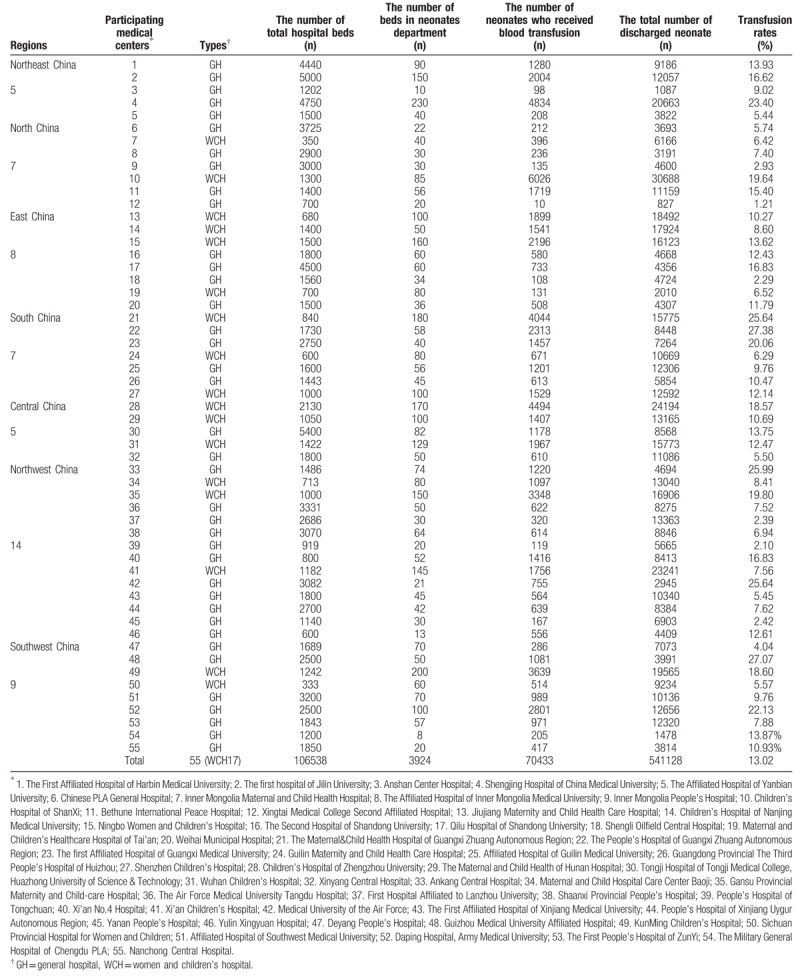
The basic information of the 55 hospitals and analysis of transfusion rates in neonates and the total number of discharged neonates between 2012 and 2016.

### 3.2 Transfusion rates in neonates

Between January 1, 2012 and December 31, 2016, the total number of discharged neonates in the 55 hospitals nationwide was 541,128, and the number of neonates who received blood transfusion was 70,433, with an average transfusion rate of 13.02%. The blood transfusion rates in hospitals of different sizes varied between 1.21% and 27.38% (Table 1). In addition, there were considerable differences in the transfusion rates between different regions. The highest transfusion rate was in the Northeast region (17.99%), whereas the lowest transfusion rate was in the Northwest region (9.74%). The transfusion rates in other regions were between 10.60% and 16.22% (Table 2).

**Table 2:**
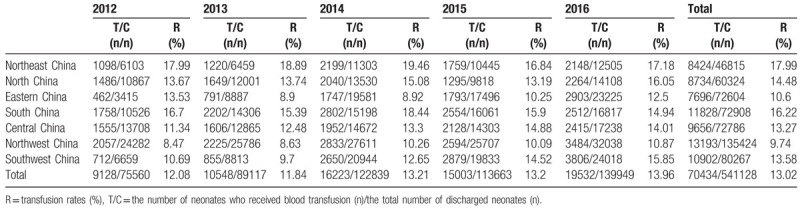
Analysis of transfusion rates in 7 regions of China.

The data were grouped into quartiles based on the number of beds in the Department of Neonatology of the hospitals (Table 3). The transfusion rate was the lowest (7.55%) in hospitals with less than 48 beds, and the highest (16.24%) in hospitals with more than 91 beds.

**Table 3:**
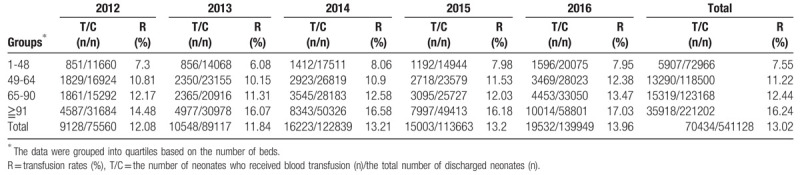
Analysis of transfusion rates in different number of beds of Department of Neonatology.

Statistical analysis by hospital type showed that the neonatal blood transfusion rate was 12.26% in general hospitals and 13.8% in women and children's hospitals. The overall blood transfusion rate in women and children's hospitals was greater than that in general hospitals (Table S1).

### 3.3 Blood component transfusion rates in neonates

Of the 55 hospitals, 39 participated in the survey on neonatal blood component transfusion rates. The number of blood transfusions between January 1, 2014 and December 31, 2016 were calculated. The total number of hospitalized neonates in the 39 hospitals within the 3-year reference period was 259,886. About 24,536 neonatal patients received red blood cell suspension transfusion (9.44%), 1717 neonatal patients received platelet transfusion (0.66%), and 12,394 neonatal patients received plasma transfusion (4.77%) (Table 4). The rates of red blood cell suspension (14.3%), plasma (6.75%), and platelet transfusions (1.41%) were highest in Northeast China. The lowest rate of red blood cell transfusion was in Southwest China (8.12%), the lowest rate of platelet transfusion was in Northwest China (0.41%), and the lowest rate of plasma transfusion was in East China (3.26%) (Table 4). The rates of red blood cell suspension and platelet transfusions in neonates were lower in general hospitals than in women and children's hospitals, whereas the rate of plasma transfusion in both hospitals was the same (Table S2).

**Table 4:**
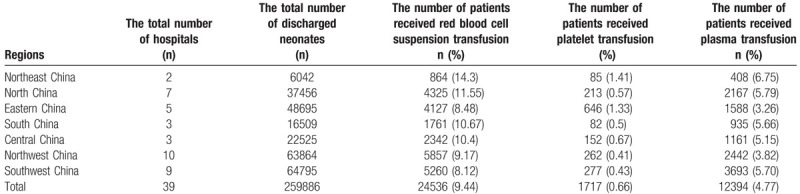
Blood component transfusion rates in neonates in different regions.

### 3.4 Spectrum of diseases in hospitalized neonates and that in hospitalized neonates treated with blood transfusion

The disease spectrum in neonates was obtained by weighting the top 10 assigned diseases in each participating hospital each year and the total number of hospitalized neonatal patients in the 55 hospitals (Table 5). Between 2012 and 2016, the top 10 diseases in hospitalized neonates were, in rank order, as follows: pneumonia, prematurity, hyperbilirubinemia, respiratory distress syndrome, asphyxia, hemolytic disease, bacterial sepsis, hypoxic-ischemic encephalopathy, aspiration pneumonia, and dysphagia. The rank order in the disease spectrum of the hospitalized neonates varied in different regions. Among the hospitalized patients in Northeast China and North China, the number of preterm neonates was the highest and ranked 1st in the spectrum. In East China, South China, Northwest China, and Southwest China, the disease with the highest rank was hyperbilirubinemia, whereas that in Central China was pneumonia. Except for Southwest China, the top three leading causes of hospitalization in other regions were prematurity, hyperbilirubinemia, and pneumonia (Table S3).

**Table 5:**
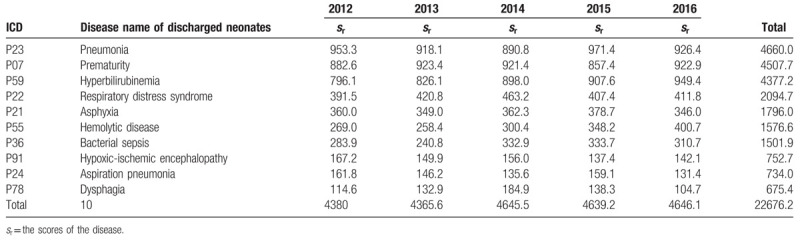
Spectrum of diseases of hospitalized neonates in the 55 hospitals (Top 10).

Data on the hospitalized neonates who received blood transfusions in the 55 hospitals were weighted, and a spectrum of diseases in neonates treated by blood transfusion was obtained (Table 6). The top 10 diseases identified in neonates treated with blood transfusion were, in rank order, as follows: prematurity, pneumonia, hyperbilirubinemia, bacterial sepsis, respiratory distress syndrome, anemia, hemolytic disease, asphyxia, hemorrhage, and necrotizing enterocolitis. The rank order of diseases in neonates treated with blood transfusion varied in different regions. The disease that ranked first in Northeast China, North China, East China, South China, and Northwest China was prematurity, and those in Southwest China and Central China were hyperbilirubinemia and pneumonia, respectively (Table S4).

**Table 6:**
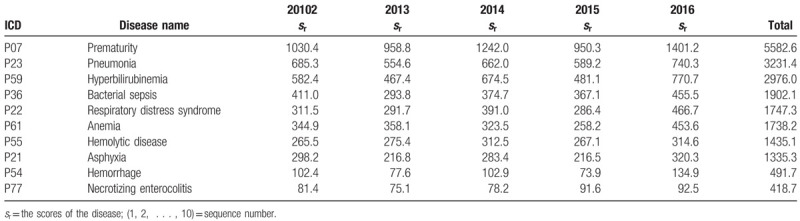
Spectrum of diseases in neonates treated by blood transfusion (Top 10).

## 4 Discussion

Recent clinical studies have found that the effect of the strongly advocated restrictive transfusion strategy on neurodevelopment remains uncertain.^[8–14]^ In the era of evidence-based medicine, transfusion strategies require the support of strong evidence from clinical research. Generally, transfusions can be considered as long as the benefits outweigh the risks. However, the evolution of transfusion strategies in preterm neonates in recent decades lacks the support from clinical evidence. The criteria for determining blood transfusion need in critically ill neonates are imprecise, and opinions are divided among neonatologists. The use of the relatively objective parameters (such as hemoglobin or hematocrit) as guidance for blood transfusion also remains controversial. Current guidelines for neonatal blood transfusions are still in contention, and references for disease spectrum or the current status of neonatal blood transfusions are lacking. For these reasons, we conducted a multicenter study on neonatal blood transfusion in China. This study aimed to investigate the changes in the disease spectra in neonates in recent years, the results of which will allow us to understand and further explore the current status of neonatal blood transfusion in China.

This study analyzed the data on hospitalized neonates and blood transfusions between January 1, 2012 and December 31, 2016 in 55 hospitals in China. The transfusion rate was 13.02%. Neonates with younger gestational ages, lower birth weights, and more severe conditions have a higher probability of receiving blood transfusion therapy. Some researchers reported that 20% to 25% of neonates in the neonatal intensive care unit (NICU) received one or more platelet transfusions,^[15]^ and a previous study showed that 532 of 952 (55.9%) very-low-birth-weight infants received blood transfusion.^[16]^ A single-center study by Zhou et al^[17]^ reported that the transfusion rate in neonates was 25.24% and that in preterm neonates was 38.68%. The present study shows that the average blood transfusion rate in the 55 hospitals nationwide is 13.03%, which is lower than the neonatal blood transfusion rates reported by Zhou et al (25.24%) and other studies (39.23%).^[18]^ This may be due to the difference in hospital size. Among the 55 hospitals included in the present study, 8 hospitals have a transfusion rate of more than 20%. The average transfusion rate in hospitals with more than 91 neonatal beds is 16.24%, which is much higher than the transfusion rate reported in hospitals with less than 48 neonatal beds (7.55%). These results show that hospitals with more neonatal beds have higher treatment capacities, more critically ill neonatal patients, and higher blood transfusion rates. This study also revealed that neonatal transfusion rates vary in different regions of China. For example, the highest transfusion rate is in the Northeast region (17.99%), and the lowest transfusion rate is in the Northwest region (9.74%). Therefore, regional factors should be considered when formulating expert consensus on neonatal transfusion.

The results of the present survey show that among neonatal transfusions, the rate of red blood cell transfusion is 9.44%, which is significantly higher than the transfusion rates of plasma and platelets. However, there are few domestic and foreign reports on blood component transfusion rates in neonates. Platelet transfusion is often used to treat thrombocytopenia of different etiologies. It has been reported that thrombocytopenia occurs in 22% of neonates in the NICU, and the risk of intracranial hemorrhage is higher in preterm neonates with thrombocytopenia. However, the platelet transfusion rate observed in our study was only 0.66%, and the underlying reason of such a low percentage warrants further investigation. In this study, the neonatal plasma transfusion rate was 4.77%. An epidemiological survey conducted by a British group showed that the plasma transfusion rate was 3.3% in hospitalized patients aged 0 to 19 years, which was lower than the transfusion rates of red blood cells and platelets.^[9]^ Neonatal transfusion of plasma and its products are mainly used to supplement coagulation factors. As the levels of coagulation factors and plasminogen in neonates are low, they are at risk for both hemorrhage and thrombosis. This survey also shows that the types of blood components transfused in neonates indicate regional differences. The rates of red blood cell suspension (14.3%), plasma (6.75%), and platelet transfusions (1.41%) were highest in the Northeast China. So when we develop the neonatal blood transfusion treatment guidelines in China in the future, we should consider the geography of China.

The results of this survey show that the top 10 diseases identified in hospitalized neonates in China were summarized at Table 5. Compared with the disease types identified in discharged patients from the Department of Neonatology in the general hospitals described in the 2016 report on the statistical analysis of medical data^[19]^ (pneumonia, prematurity, hyperbilirubinemia, asphyxia, respiratory distress syndrome, low birth weight, bacterial sepsis [septicemia], diarrhea, and transient tachypnea of the newborn). The results were basically consistent with the present study. The present study shows that respiratory diseases account for the largest number of diseases, with 4 (33.40%) of the top 10 diseases being respiratory diseases (pneumonia, respiratory distress syndrome, asphyxia, and aspiration pneumonia). This study also showed that preterm neonates account for 12.09% of all hospitalized neonates, which is slightly higher than the 11.10% incidence rate of preterm neonates reported in the “Global Action Report on Preterm Birth” in 2012,^[20]^ and is significantly higher than the 7.8% preterm birth rate reported in the large-scale epidemiological survey on preterm neonates conducted in China in 2005.^[21]^

Between 2012 and 2016, the top 10 diseases identified in hospitalized neonates treated with blood transfusion in 55 hospitals in China were summarized at Table 6. The understanding of the disease spectrum in neonates who received blood transfusions can assist the formulation of expert consensus on neonatal transfusions, but there are few domestic and foreign reports on the spectrum of diseases in neonates treated with blood transfusion. Our study has also shown that the top 10 diseases in hospitalized neonates only partially overlap with the top 10 diseases in those treated with blood transfusion. In other words, not all of the top 10 diseases identified in hospitalized neonates require transfusion therapy during hospitalization. Limitation of the study: This is a pilot study for a national multicenter cohort study on neonatal blood transfusion. The data collected do not include detailed data on the neonatal blood transfusion cases and transfusion data from neonates in the NICU of each hospital.

## 5 Conclusion

The study can provide supporting data for the next phase of study conducted on Chinese neonatal cohort and provide technical data and theoretical evidence for the development of neonatal blood transfusion strategies in clinical settings.

## Acknowledgments

Thanks to the following people of 55 hospitals for their contributions to this article, in no particular order. Anyan Deng BS, Chong Zhang PhD, Danni Zhong PhD, Dong Zhou PhD, Fang Han PhD, Fang Liu PhD, Feng Chen MD, Fenghua Liu BS, Hailan Li BS, Hasitana MD, Heqin Li BS, Hongbing Hu MD, Hua Mei MD, Huiqing Sun PhD, Jianchun Lv BS, Jiang Xue MD, Jie Xiao MD, Juan Wang MD, Kexuan Qu MD, Lijun Mei BS, Lingli Miao BS, Long Chen PhD, Long Li BS, Maoqiong Chen MD, Ping Zheng BS, Qian Hou BS, Qiang Feng BS, Qin Lv MD, Qiuju Liu PhD, Rongxuan Wei MD, Ru Lin MD, Shengchao Jin PhD, Shuping Dai MD, Shurong Li BS, Sihua Yi PhD, Tao Peng PhD,Tianhua Jiang BS, Wen Yin PhD, Xiao Zhang MD, Xiaoliang Zeng BS, Xiling Li BS, Xuezhen Song BS, Xun Jiang PhD, Yang yu PhD, Yi Wei PhD, Yingying Guo MD, Yuanshuai Huang PhD, Zhenai Jin PhD.

## Author contributions

**Conceptualization:** Jiang-cun Yang, Zhen-ai Jin.

**Data curation:** Yang Sun, Qin Zhang.

**Formal analysis:** Ting Ma, Wen-hua Wang.

**Investigation:** Yang Sun, Ting Ma.

**Methodology:** Jiang-cun Yang.

**Writing – original draft:** Yang Sun, Ting Ma.

**Writing – review & editing:** Jiang-cun Yang, Ting Ma.

## Supplementary Material

Supplemental Digital Content

## Supplementary Material

Supplemental Digital Content

## Supplementary Material

Supplemental Digital Content

## Supplementary Material

Supplemental Digital Content
