# Supplementary material for: Transfusion rates and disease spectrum in neonates treated with blood transfusion in China
Source: Medicine (Baltimore). 2020 May 1;99(18):e19961. doi: 10.1097/MD.0000000000019961 (PMC7440345; doi:10.1097/MD.0000000000019961)
Supplement: Supplemental Digital Content [file medi-99-e19961-s001.docx]

Supplementary Table 1 Analysis of transfusion rates in general hospitals and women and children’s hospitals

|  | 2012 | | 2013 | | 2014 | | 2015 | | 2016 | | Total | |
| --- | --- | --- | --- | --- | --- | --- | --- | --- | --- | --- | --- | --- |
|  | T/C | R | T/C | R | T/C | R | T/C | R | T/C | R | T/C | R |
|  | (n/n) | （%） | (n/n) | （%） | (n/n) | （%） | (n/n) | （%） | (n/n) | （%） | (n/n) | （%） |
| General hospital | 4476/39207 | 11.42 | 5048/45259 | 11.15 | 7894/63095 | 12.51 | 7255/57316 | 12.66 | 9106/70694 | 12.88 | 33779/275571 | 12.26 |
| Women and children’s hospital | 4652/36353 | 12.8 | 5500/43858 | 12.54 | 8329/59744 | 13.94 | 7748/56347 | 13.75 | 10426/69255 | 15.05 | 36655/265557 | 13.8 |
| Total | 9128/75560 | 12.08 | 10548/89117 | 11.84 | 16223/122839 | 13.21 | 15003/113663 | 13.2 | 19532/139949 | 13.96 | 70434/541128 | 13.02 |
