# Supplementary material for: Transfusion rates and disease spectrum in neonates treated with blood transfusion in China
Source: Medicine (Baltimore). 2020 May 1;99(18):e19961. doi: 10.1097/MD.0000000000019961 (PMC7440345; doi:10.1097/MD.0000000000019961)
Supplement: Supplemental Digital Content [file medi-99-e19961-s002.docx]

Supplementary Table 2 Compare of blood component transfusion rates in neonates between general hospitals and women and children’s hospitals

| Types | The total number of hospital | The number of discharged neonates  （n） | The number of patients received red blood cell suspension transfusion | The number of patients received platelet transfusion | The number of patients received plasma transfusion |
| --- | --- | --- | --- | --- | --- |
|  | (n) |  | n(%) | n(%) | n(%) |
| General hospital | 28 | 131803 | 11005(8.35) | 810(0.61) | 6283(4.77) |
| Women and children’s hospital | 11 | 128083 | 13531(10.56) | 907(0.71) | 6111(4.77) |
| Total | 39 | 259886 | 24536(9.44) | 1717(0.66) | 12394(4.77) |
