# Supplementary material for: Transfusion rates and disease spectrum in neonates treated with blood transfusion in China
Source: Medicine (Baltimore). 2020 May 1;99(18):e19961. doi: 10.1097/MD.0000000000019961 (PMC7440345; doi:10.1097/MD.0000000000019961)
Supplement: Supplemental Digital Content [file medi-99-e19961-s003.docx]

Supplementary Table 3 Spectrum of diseases of hospitalized neonates in different regions (Top 10)

|  | ICD | Disease name of hospitalized neonates | \| Total \| \| --- \| \| s_r_ \| | \| 2012 \| \| --- \| \| s_r_ \| | \| 2013 \| \| --- \| \| s_r_ \| | \| 2014 \| \| --- \| \| s_r_ \| | \| 2015 \| \| --- \| \| s_r_ \| | \| 2016 \| \| --- \| \| s_r_ \| |
| --- | --- | --- | --- | --- | --- | --- | --- | --- | --- | --- | --- | --- | --- | --- | --- | --- | --- | --- | --- | --- |
| Northeast China | P07 | prematurity | 880.2（1） | 204.1（1） | 181.9（1） | 183.6（1） | 139.4（1） | 171.1（1） |
|  | P59 | hyperbilirubinemia | 307.4（2） | 42.9（4） | 63.7（3） | 57.5（2） | 71.4（2） | 71.9（2） |
|  | P23 | pneumonia | 296.4（3） | 69.7（2） | 59.2（4） | 57（3） | 61.2（3） | 49.4（3） |
|  | P22 | respiratory distress syndrome | 259.1（4） | 56.5（3） | 64.6（2） | 48.2（4） | 49.1（5） | 40.5（4） |
|  | P21 | asphyxia | 199.5（5） | 39.4（6） | 37.8（5） | 42.8（5） | 50.9（4） | 28.7（6） |
|  | P91 | hypoxic-ischemic encephalopathy | 160.9（6） | 36.7（7） | 31.5（6） | 28.9（6） | 30.3（6） | 33.5（5） |
|  | P05 | small for date infant | 109.3（7） | 40.6（5） | 26.9（7） | 16.4（9） | 13.1（10） | 12.3（10） |
|  | P36 | bacterial sepsis | 108.6（8） | 23.9（9） | 22.8（8） | 22.3（7） | 19.2（8） | 20.4（8） |
|  | P28 | respiratory failure | 108.5（9） | 28.5（8） | 18.7（9） | 18.2（8） | 21.1（7） | 21.9（7） |
|  | P55 | hemolytic disease | 51.8（10） | 4.3（10） | 3（10） | 8.4（10） | 15.9（9） | 20.1（9） |
| North China | P07 | prematurity | 698.7（1） | 152.8（1） | 144.4（1） | 144.3（1） | 123（1） | 134.2（1） |
|  | P23 | pneumonia | 485（2） | 103.3（2） | 101.7（2） | 106.5（2） | 82.9（2） | 90.6（2） |
|  | P59 | hyperbilirubinemia | 284.1（3） | 40.2（5） | 56.5（4） | 68.5（3） | 54.9（3） | 64（3） |
|  | P22 | respiratory distress syndrome | 265.2（4） | 59.2（3） | 65.5（3） | 62（4） | 31.5（6） | 47（4） |
|  | P36 | bacterial sepsis | 210.9（5） | 50.2（4） | 48.2（5） | 46.3（5） | 33.3（5） | 32.8（6） |
|  | P21 | asphyxia | 185.1（6） | 38.1（6） | 23.2（9） | 30.1（7） | 49.1（4） | 44.6（5） |
|  | P61 | anemia | 148.7（7） | 26.8（8） | 35.9（6） | 33.6（6） | 24.7（7） | 27.6（7） |
|  | P39 | intrauterine infection | 119.6（8） | 22.9（9） | 28.4（7） | 23.3（8） | 17.3（8） | 27.6（8） |
|  | P78 | dysphagia | 81.2（9） | 21.3（10） | 17.7（10） | 22.8（9） | 6.5（9） | 12.9（9） |
|  | P54 | hemorrhage | 80.4（10） | 36.7（7） | 28.1（8） | 15.6（10） | （10） | （10） |
| Eastern China | P59 | hyperbilirubinemia | 666.9（1） | 99.2（1） | 104.2（2） | 161.4（1） | 146.8（1） | 155.2（1） |
|  | P23 | pneumonia | 574.2（2） | 72.5（3） | 69.7（3） | 137.4（2） | 145.3（2） | 149.4（2） |
|  | P07 | prematurity | 496.1（3） | 83.8（2） | 109.3（1） | 101.9（3） | 91.4（3） | 109.7（3） |
|  | P36 | bacterial sepsis | 255.6（4） | 27（8） | 26.7（7） | 64.6（4） | 70.2（4） | 67（4） |
|  | P21 | asphyxia | 253.4（5） | 50.2（5） | 41.9（6） | 58.5（6） | 61.7（5） | 41.1（7） |
|  | P22 | respiratory distress syndrome | 214.2（6） | 33.5（7） | 9.3（10） | 61.5（5） | 48（6） | 62（5） |
|  | P55 | hemolytic disease | 163.5（7） | 14.6（9） | 15.2（9） | 28.8（8） | 47.8（7） | 57（6） |
|  | P01 | impact of maternal pregnancy complications | 145.9（8） | 52.2（4） | 48.3（5） | （10） | 31.2（8） | 14.2（9） |
|  | P96 | specified conditions originating perinatal period | 142.7（9） | 45.6（6） | 54.4（4） | 42.6（7） | （10） | （10） |
|  | P24 | aspiration pneumonia | 114.8（10） | 6.5（10） | 24.5（8） | 26.7（9） | 31.2（9） | 25.9（8） |
| South China | P59 | hyperbilirubinemia | 635.2（1） | 118.7（1） | 133.4（1） | 120.5（1） | 139.7（2） | 123（3） |
|  | P07 | prematurity | 616（2） | 86.4（3） | 127.1（2） | 120.4（2） | 153.9（1） | 128.2（1） |
|  | P23 | pneumonia | 567.8（3） | 87.6（2） | 112.9（3） | 104.3（3） | 139.3（3） | 123.7（2） |
|  | P55 | hemolytic disease | 215.1（4） | 43.1（4） | 35.8（6） | 39.1（6） | 54.7（4） | 42.4（4） |
|  | P22 | respiratory distress syndrome | 208.7（5） | 27.5（7） | 49.7（4） | 45.2（5） | 47.6（5） | 38.8（5） |
|  | P78 | dysphagia | 175.2（6） | 26.2（8） | 45.4（5） | 46（4） | 35.4（6） | 22.3（7） |
|  | P24 | aspiration pneumonia | 154（7） | 31.9（5） | 30.5（7） | 25.1（7） | 33.1（7） | 33.3（6） |
|  | P36 | bacterial sepsis | 105.3（8） | 29.8（6） | 21.9（8） | 19.8（8） | 19.2（9） | 14.6（9） |
|  | P96 | specified conditions originating perinatal period | 91.2（9） | 19.3（10） | 17.7（10） | 18.4（9） | 19.7（8） | 16.1（8） |
|  | P39 | intrauterine infection | 75.1（10） | 20.5（9） | 19.1（9） | 15.7（10） | 8（10） | 11.7（10） |
| Central China | P23 | pneumonia | 945.8（1） | 215.8（1） | 200.3（1） | 163.7（1） | 191.8（1） | 174.2（1） |
|  | P59 | hyperbilirubinemia | 638.3（2） | 137.5（2） | 109.4（2） | 135.8（2） | 121.3（2） | 134.4（2） |
|  | P07 | prematurity | 396.2（3） | 104（3） | 68.8（3） | 70.2（4） | 75.7（4） | 77.4（3） |
|  | P36 | bacterial sepsis | 368（4） | 64（4） | 50.1（4） | 85.1（3） | 95.8（3） | 72.9（4） |
|  | P21 | asphyxia | 190.4（5） | 41.1（5） | 48.1（5） | 32.8（6） | 30.6（7） | 37.8（6） |
|  | P55 | hemolytic disease | 169（6） | 30.4（8） | 30.5（7） | 37.4（5） | 28（8） | 42.7（5） |
|  | P22 | respiratory distress syndrome | 168.4（7） | 33.3（7） | 31.5（6） | 31.8（7） | 37.2（5） | 34.7（7） |
|  | P91 | hypoxic-ischemic encephalopathy | 138.2（8） | 34（6） | 21.6（8） | 22.1（9） | 30.7（6） | 29.8（8） |
|  | P78 | dysphagia | 74.1（9） | 24.6（9） | 6.7（10） | 24.5（8） | 15.5（9） | 2.8（10） |
|  | P70 | endocrinological and metabolic disease | 64.9（10） | 10.6（10） | 7.4（9） | 14.8（10） | 10（10） | 22（9） |
| Northwest China | P59 | hyperbilirubinemia | 1125.9（1） | 248.9（2） | 232.1（2） | 202.9（2） | 219.6（1） | 222.5（2） |
|  | P07 | prematurity | 1107.5（2） | 207（3） | 224.6（3） | 227.9（1） | 208.2（2） | 239.8（1） |
|  | P23 | pneumonia | 1103.8（3） | 281.3（1） | 235.2（1） | 180.4（3） | 205.8（3） | 201.1（3） |
|  | P21 | asphyxia | 547.8（4） | 109.7（4） | 110.5（4） | 113.8（4） | 101.1（5） | 112.7（4） |
|  | P55 | hemolytic disease | 538.5（5） | 103.5（5） | 100.2（5） | 102.3（5） | 120.4（4） | 112（5） |
|  | P22 | respiratory distress syndrome | 341.1（6） | 70.8（8） | 73.7（6） | 71.7（7） | 55.9（7） | 68.9（7） |
|  | P24 | aspiration pneumonia | 332.7（7） | 82.9（6） | 73.5（7） | 71.8（6） | 68（6） | 36.5（9） |
|  | P36 | bacterial sepsis | 305.9（8） | 67.9（10） | 60.9（9） | 52.9（8） | 54.1（8） | 70（6） |
|  | P61 | anemia | 274.1（9） | 68.3（9） | 67.6（8） | 49.1（9） | 46.6（9） | 42.5（8） |
|  | P91 | hypoxic-ischemic encephalopathy | 213.4（10） | 73.1（7） | 56.9（10） | 47.1（10） | 22.7（10） | 13.5（10） |
| Southwest  China | P59 | hyperbilirubinemia | 719.4（1） | 108.7（3） | 126.9（2） | 151.4（1） | 154（1） | 178.5（1） |
|  | P23 | pneumonia | 686.9（2） | 123.1（1） | 139.1（1） | 141.5（3） | 145.2（2） | 138（2） |
|  | P22 | respiratory distress syndrome | 638（3） | 110.7（2） | 126.6（3） | 142.7（2） | 138.1（3） | 119.9（3） |
|  | P55 | hemolytic disease | 369.9（4） | 66.5（5） | 60.6（6） | 78.2（4） | 71.5（4） | 93.1（4） |
|  | P21 | asphyxia | 351.8（5） | 68.8（4） | 78.2（4） | 66.9（6） | 67.8（5） | 70.2（5） |
|  | P07 | prematurity | 313（6） | 44.5（6） | 67.2（5） | 73（5） | 66（6） | 62.4（6） |
|  | P78 | dysphagia | 207.3（7） | 13.4（10） | 32.9（8） | 58.6（7） | 59.6（7） | 42.6（7） |
|  | P36 | bacterial sepsis | 147.8（8） | 21.1（9） | 10.2（10） | 41.7（8） | 41.8（8） | 32.9（8） |
|  | P77 | necrotizing enterocolitis | 119.7（9） | 28.6（7） | 39.6（7） | 21.7（9） | 29.7（9） | （10） |
|  | P24 | aspiration pneumonia | 87.2（10） | 21.7（8） | 14.1（9） | 11.4（10） | 15（10） | 24.9（9） |

s_r_: the scores of the disease，（1,2，---10）：sequence number
