# Supplementary material for: Transfusion rates and disease spectrum in neonates treated with blood transfusion in China
Source: Medicine (Baltimore). 2020 May 1;99(18):e19961. doi: 10.1097/MD.0000000000019961 (PMC7440345; doi:10.1097/MD.0000000000019961)
Supplement: Supplemental Digital Content [file medi-99-e19961-s004.docx]

Supplementary Table 4 Spectrum of diseases in neonates treated by blood transfusion in different regions（Top 10）

| Regions | ICD | Disease name |  | \| Total \| \| --- \| | \| 20102 \| \| --- \| \| s_r_ \| | \| 20102 \| \| --- \| \| s_r_ \| | \| 20102 \| \| --- \| \| s_r_ \| | \| 20102 \| \| --- \| \| s_r_ \| | \| 20102 \| \| --- \| \| s_r_ \| |
| --- | --- | --- | --- | --- | --- | --- | --- | --- | --- | --- | --- | --- | --- | --- | --- | --- | --- | --- | --- | --- |
| Northeast China | P07 | prematurity |  | 1386.8（1） | 202.3（1） | 331.8（1） | 360.6（1） | 209.9（1） | 282.2（1） |
|  | P59 | hyperbilirubinemia |  | 452.2（2） | 87.1（3） | 118（2） | 102.4（2） | 60.6（2） | 84（2） |
|  | P61 | anemia |  | 411（3） | 92（2） | 100.3（3） | 81.1（3） | 55.1（3） | 82.4（3） |
|  | P23 | pneumonia |  | 226.3（4） | 59.2（4） | 47.5（7） | 52.3（4） | 32.3（4） | 34.9（7） |
|  | P36 | bacterial sepsis |  | 221.5（5） | 55.1（5） | 53.5（6） | 45.4（5） | 29.4（5） | 38（5） |
|  | P22 | respiratory distress syndrome |  | 217.2（6） | 44.2（6） | 67.6（4） | 37.4（7） | 29.4（6） | 38.7（4） |
|  | P55 | hemolytic disease |  | 182.8（7） | 23.9（8） | 58.1（5） | 42.3（6） | 21.8（7） | 36.7（6） |
|  | P05 | small for date infant |  | 116.2（8） | （10） | 46.1（8） | 30.1（8） | 20.1（8） | 19.9（8） |
|  | P28 | respiratory failure |  | 109.2（9） | 26.2（7） | 27（9） | 22.7（9） | 14.3（9） | 19.1（9） |
|  | P54 | hemorrhage |  | 71.6（10） | 17.5（9） | 17.6（10） | 14.6（10） | 9.5（10） | 12.5（10） |
| North China | P07 | prematurity |  | 440.7（1） | 59.1（1） | 45.1（1） | 44.8（1） | 101.8（1） | 189.9（1） |
|  | P21 | asphyxia |  | 210.9（2） | 34.6（2） | 31.3（2） | 34.1（2） | 44.3（3） | 66.5（4） |
|  | P36 | bacterial sepsis |  | 179.6（3） | 22.5（6） | 21.3（5） | 20.5（6） | 42.1（4） | 73.2（3） |
|  | P23 | pneumonia |  | 158.9（4） | 5.4（9） | 4.8（7） | 5.7（8） | 49.2（2） | 94（2） |
|  | P22 | respiratory distress syndrome |  | 137（5） | 28.7（4） | 25.2（4） | 24.4（3） | 24.5（6） | 34.2（7） |
|  | P61 | anemia |  | 127.2（6） | 11.3（7） | 9.3（6） | 12.5（7） | 28.9（5） | 65.2（5） |
|  | P59 | hyperbilirubinemia |  | 108.9（7） | 29.8（3） | 26.7（3） | 21.7（5） | 11.7（8） | 19（10） |
|  | P55 | hemolytic disease |  | 76（8） | 22.9（5） | 2.2（9） | 4.8（9） | 1.1（10） | 45.1（6） |
|  | P26 | pulmonary hemorrhage |  | 57.5（9） | （10） | 0.4（10） | 23（4） | 11.1（9） | 22.9（8） |
|  | P77 | necrotizing enterocolitis |  | 49.6（10） | 7.7（8） | 2.4（8） | 1.2（10） | 18.8（7） | 19.5（9） |
| East China | P07 | prematurity |  | 435.3（1） | 7.3（8） | 10.8（7） | 134（1） | 85.9（1） | 197.2（1） |
|  | P23 | pneumonia |  | 322.8（2） | 52.1（1） | 49.9（2） | 60.2（3） | 69.7（2） | 91（3） |
|  | P59 | hyperbilirubinemia |  | 283.3（3） | 27.4（6） | 19.6（6） | 60.3（2） | 57.7（4） | 118.2（2） |
|  | P36 | bacterial sepsis |  | 252（4） | 34.6（5） | 37.9（3） | 51.9（5） | 60.4（3） | 67.1（6） |
|  | P21 | asphyxia |  | 225.8（5） | 38.9（3） | 21.9（5） | 54.9（4） | 40.8（6） | 69.2（5） |
|  | P61 | anemia |  | 225.6（6） | 36.3（4） | 50.2（1） | 45.6（6） | 28.4（8） | 65.1（7） |
|  | P55 | hemolytic disease |  | 203（7） | 43.3（2） | 29.2（4） | 29.4（8） | 46.7（5） | 54.4（8） |
|  | P22 | respiratory distress syndrome |  | 168.5（8） | 20.2（7） | 6.3（8） | 30.8（7） | 31.6（7） | 79.5（4） |
|  | P39 | intrauterine infection |  | 44.1（9） | 0（9） | 0（9） | 13（9） | 8.7（10） | 22.5（10） |
|  | P78 | dysphagia |  | 42.7（10） | （10） | （10） | 5.9（10） | 12.3（9） | 24.5（9） |
| South China | P07 | prematurity |  | 1038.8（1） | 236.3（1） | 232.1（1） | 222.9（1） | 186.5（1） | 161.1（1） |
|  | P59 | hyperbilirubinemia |  | 541（2） | 143.3（2） | 116（2） | 100.6（2） | 65.4（2） | 115.7（2） |
|  | P23 | pneumonia |  | 395.4（3） | 82（4） | 80.2（5） | 95.6（3） | 60.4（4） | 77.1（4） |
|  | P61 | anemia |  | 303.8（4） | 72.9（5） | 97.4（3） | 50.6（5） | 33.9（5） | 49（5） |
|  | P55 | hemolytic disease |  | 273（5） | 37.7（6） | 92.7（4） | 49.2（6） | 63.3（3） | 30（7） |
|  | P22 | respiratory distress syndrome |  | 260.8（6） | 10.6（10） | 36（10） | 95.1（4） | 31.1（7） | 87.9（3） |
|  | P36 | bacterial sepsis |  | 235.7（7） | 88.6（3） | 39.4（9） | 32.6（8） | 30（8） | 45.2（6） |
|  | P21 | asphyxia |  | 154（8） | 21.1（9） | 47.8（6） | 42.5（7） | 31.2（6） | 11.4（9） |
|  | P60 | diffuse intravascular coagulation |  | 112.9（9） | 25.4（8） | 43.1（7） | 20.5（9） | 10.9（9） | 13.1（8） |
|  | P24 | aspiration pneumonia |  | 101.1（10） | 25.7（7） | 42.9（8） | 14.1（10） | 10.4（10） | 7.9（10） |
| Central China | P23 | pneumonia |  | 1108.5（1） | 270.3（1） | 235.8（1） | 200.1（1） | 188（1） | 214.3（1） |
|  | P07 | prematurity |  | 571.2（2） | 188.3（2） | 113（2） | 84.2（4） | 74.9（3） | 110.8（3） |
|  | P36 | bacterial sepsis |  | 478.3（3） | 113.2（3） | 87（3） | 97.1（3） | 95.3（2） | 85.8（4） |
|  | P59 | hyperbilirubinemia |  | 471.4（4） | 111.6（4） | 74.1（4） | 108.6（2） | 60.9（4） | 116.1（2） |
|  | P22 | respiratory distress syndrome |  | 354（5） | 70.9（5） | 73.9（5） | 70.5（5） | 56.6（5） | 82.1（5） |
|  | P21 | asphyxia |  | 240.1（6） | 65.5（6） | 45（6） | 57（6） | 31.4（6） | 41.1（6） |
|  | P55 | hemolytic disease |  | 124.3（7） | 24.5（7） | 36（7） | 36.1（7） | 24.1（7） | 3.6（10） |
|  | P54 | hemorrhage |  | 81.3（8） | 17.3（8） | 14.4（8） | 19.2（8） | 8.7（9） | 21.8（7） |
|  | P05 | small for date infant |  | 65.9（9） | 14.8（9） | 12.3（9） | 13（10） | 10.2（8） | 15.5（8） |
|  | P61 | anemia |  | 48.6（10） | 10.8（10） | 8.2（10） | 14.9（9） | 5.8（10） | 8.9（9） |
| Northwest China | P07 | prematurity |  | 1122.8（1） | 272.4（1） | 176.7（1） | 234.1（1） | 170.4（1） | 269.2（1） |
|  | P23 | pneumonia |  | 531.9（2） | 144（2） | 89.4（2） | 117.3（2） | 92.5（2） | 88.7（4） |
|  | P61 | anemia |  | 506.6（3） | 101.6（5） | 76.7（3） | 86（4） | 90.2（3） | 152.1（2） |
|  | P59 | hyperbilirubinemia |  | 451.9（4） | 128.9（3） | 75.6（4） | 92.7（3） | 59（5） | 95.8（3） |
|  | P55 | hemolytic disease |  | 396.1（5） | 110.4（4） | 56（5） | 79（5） | 65.5（4） | 85.2（5） |
|  | P36 | bacterial sepsis |  | 300.3（6） | 64.3（8） | 49.2（6） | 72（6） | 42.7（7） | 72.1（6） |
|  | P22 | respiratory distress syndrome |  | 267.6（7） | 72.1（6） | 39.8（7） | 55.5（7） | 45.2（6） | 54.9（8） |
|  | P21 | asphyxia |  | 228.1（8） | 71.9（7） | 38.2（8） | 40.1（9） | 22.6（10） | 55.1（7） |
|  | P77 | necrotizing enterocolitis |  | 194.8（9） | 45.8（9） | 31.1（9） | 46.3（8） | 30.3（8） | 41.3（10） |
|  | P54 | hemorrhage |  | 168.4（10） | 44.8（10） | 25.2（10） | 24.9（10） | 28.9（9） | 44.6（9） |
| Southwest China | P59 | hyperbilirubinemia |  | 667.4（1） | 54.4（4） | 37.3（4） | 188.1（1） | 165.7（1） | 221.9（1） |
|  | P07 | prematurity |  | 587.1（2） | 64.7（3） | 49.3（1） | 161.4（2） | 120.9（2） | 190.9（2） |
|  | P23 | pneumonia |  | 487.7（3） | 72.3（1） | 46.9（2） | 130.9（3） | 97（3） | 140.5（3） |
|  | P22 | respiratory distress syndrome |  | 342.1（4） | 64.7（2） | 42.8（3） | 77.3（4） | 68（4） | 89.3（4） |
|  | P21 | asphyxia |  | 248.6（5） | 47.6（5） | 31.8（5） | 48.6（8） | 45.1（7） | 75.5（6） |
|  | P36 | bacterial sepsis |  | 234.6（6） | 32.8（6） | 5.4（7） | 55.3（7） | 67.2（5） | 74（7） |
|  | P78 | dysphagia |  | 203.2（7） | 0.9（10） | 1.1（10） | 61.4（6） | 63.7（6） | 76.2（5） |
|  | P55 | hemolytic disease |  | 179.8（8） | 2.8（8） | 1.2（9） | 71.7（5） | 44.5（8） | 59.6（8） |
|  | P61 | anemia |  | 115.4（9） | 20（7） | 15.9（6） | 32.8（10） | 15.9（10） | 30.8（10） |
|  | P54 | hemorrhage |  | 98.8（10） | 2.8（9） | 3.6（8） | 33.9（9） | 17.4（9） | 41.1（9） |

s_r_: the scores of the disease，（1,2，---10）：sequence number
